# Building a comprehensive mentoring academy for schools of health

**DOI:** 10.1017/cts.2019.406

**Published:** 2019-08-28

**Authors:** Julie B. Schweitzer, Julie A. Rainwater, Hendry Ton, Rebeca E. Giacinto, Candice A. M. Sauder, Frederick J. Meyers

**Affiliations:** 1 Department of Psychiatry and MIND Institute, University of California, Davis School of Medicine, Sacramento, CA, USA; 2 Clinical and Translational Science Center, University of California, Davis School of Medicine, Sacramento, CA, USA; 3 Department of Psychiatry and Office of the Dean, University of California, Davis School of Medicine, Sacramento CA, USA; 4 Department of Surgery, University of California, Davis School of Medicine, Sacramento, CA, USA; 5 Department of Internal Medicine, Division of Hematology and Oncology, Center for Precision Medicine, University of California, Davis School of Medicine, Sacramento, CA, USA

**Keywords:** Workforce development, mentor training, goal setting, individual development plans, dissemination and implementation

## Abstract

Formal mentoring programs are increasingly recognized as critical for faculty career development. We describe a mentoring academy (MA) developed for faculty across tracks (i.e., researchers, clinicians, educators) within a “school of health” encompassing schools of medicine and nursing. The program is anchored dually in a clinical and translational science center and a school of health. The structure includes the involvement of departmental and center mentoring directors to achieve widespread uptake and oversight. A fundamental resource provided by the MA includes providing workshops to enhance mentoring skills. Initiatives for junior faculty emphasize establishing and maintaining strong mentoring relationships and implementing individual development plans (IDPs) for career planning. We present self-report data on competency improvement from mentor workshops and data on resources and barriers identified by junior faculty (*n* = 222) in their IDPs. Mentors reported statistically significantly improved mentoring competency after workshop participation. Junior faculty most frequently identified mentors (61%) and collaborators (23%) as resources for goal attainment. Top barriers included insufficient time and time-management issues (57%), funding limitations (18%), work–life balance issues (18%), including inadequate time for self-care and career development activities. Our MA can serve as a model and roadmap for providing resources to faculty across traditional tracks within medical schools.

## Introduction

Mentoring, formal and deliberate guidance in career development planning, is recognized as critical for junior faculty to achieve success in academic health centers [1,2–4]. Indeed, proteges with greater support for mentoring report higher satisfaction within their institution than those without mentoring resources [5]. The majority of formal faculty mentoring programs focus on increasing success for faculty members who are primarily devoted to research careers [2,3,6–10]; however, clinicians and educators constitute a growing percentage of faculty members in academic health systems [11–15] and are also in need of formal career development mentoring.

We developed and launched a mentoring program, the mentoring academy (MA), to recognize and advance excellence in mentoring, as well as develop mentoring skills informed by best practices. Thus, from its inception, the MA acknowledged and addressed the need to reorient the culture of the institution to support, reward, and enhance quality mentoring in the human health sciences across all faculty tracks. Leadership and senior faculty envisioned that by building cadres of skilled mentors, it would facilitate the development of the next generation of independent, highly successful academic faculty and advance the overall mission of our School of Medicine (SOM). As the School of Nursing at (SON) was developing during this time period, the SON increasingly participated in the MA, thus broadening the scope even more. This article describes how we developed our mentoring program to serve a wide variety of faculty members across different academic tracks. We also present data on two of its primary resources that were initiated to change the culture, workshops on mentoring (Initiative I), and information derived from the primary career planning tool, the individual development plan (IDP), a process that was expected for junior faculty in the health system (Initiative II).

## MA Structure

**Fig. 1.**
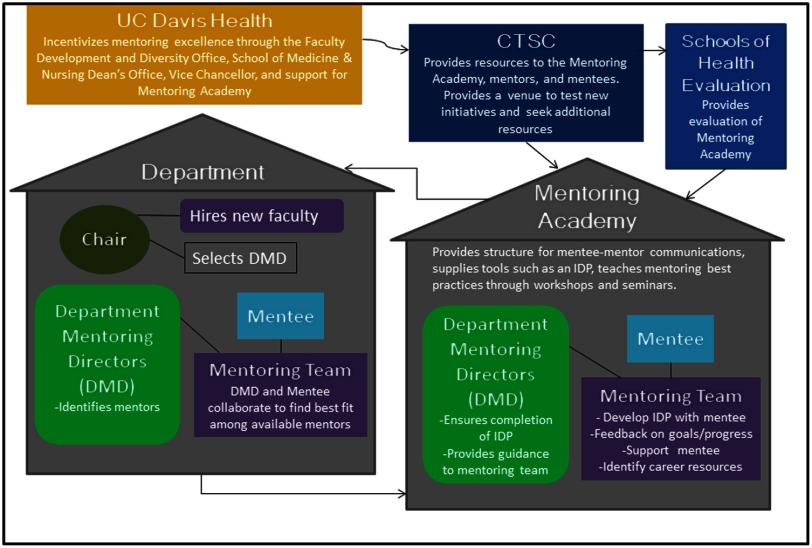
Structure and function of mentoring academy and its relationship with junior faculty members. CTSC, Clinical and Translational Science Center; IDP, individual development plans.

The MA originated, simultaneously, in two different “homes” on our health system campus (see Fig. 1). One home was the National Institutes of Health (NIH)-funded Clinical and Translational Science Center (CTSC), with an emphasis on mentoring practices for research trainees and the other “home” was the dean’s office of the schools of health (SOM & SON), with an emphasis on mentoring practices for faculty in all tracks and ranks. The CTSC mentoring program served as an incubator to develop and refine many of the practices for the broader MA. Gradually, mentoring practices from the CTSC were integrated into the broader MA, with ongoing adjustments to meet the diverse needs of faculty across academic tracks. Eventually, the MA was absorbed into a newly developed, robust, faculty development and diversity office serving the SOM and SON. Mentees include all assistant and newly appointed associate professors as well as any faculty member requesting a mentoring team. Mentors of CTSC scholars are required to participate in the mentoring workshops.

A critical component of the MA includes departmental and center resources via departmental mentoring directors (DMDs) and center mentoring directors (CMDs). These directors provide an administrative and executive link to ensure that a functioning mentoring team is in place for each junior faculty member. The DMD and CMD roles include: (1) assisting new faculty in identifying potential mentors and guiding them to resources; (2) facilitating and tracking IDP completion; (3) implementing departmental mentoring activities; (4) facilitate faculty engagement in MA activities and resources. The CMDs partner with DMDs for those mentees having a significant center association. DMDs/CMDs are provided modest financial incentives for their roles. Compensation is divided between support from the Dean’s office and the home department or center.

There are two levels of membership in the MA, General Member and Master Mentor levels. To qualify as a general member, the faculty member must apply to the MA for membership, have achieved associate (advanced) or full professor rank, participated in a minimum of two workshops on mentoring, and have experience in mentoring others. Additionally, the faculty member’s department chair must send a recommendation affirming the applicant’s skills and experience as a mentor and confirm that there are no reservations about the faculty member’s skills or history as a mentor. In order to build an “Academy” of mentors, the Master Mentor track was also created. We considered the Master’s level status as critical to signify a change in the institution’s culture by raising the recognition and reward for mentoring, with those achieving unique dedication and skill in mentoring invited into the Academy at this elevated level. The initial group of Master Mentors was involved in designing the structure, guidelines, and selection of resources. Prospective Master Mentor members are invited to apply for the Master’s level status and are expected to have achieved an advanced academic rank, present evidence of a sustained track record of excellence in mentoring, have externally recognized mentoring expertise (e.g., local or national mentoring award) or be a director of a funded training program, participated in all five MA workshops on mentoring, obtain recommendation from their department chair, and have approval by the other currently active Master Mentors. The Master Mentors must also commit to providing service to the MA after they have been awarded the Master Mentor status.

Mentees are expected to take an active role in the mentoring process [3]. We instituted two critical requirements to facilitate the career development process for all junior faculty: (1) All junior faculty are required to have a mentoring team; (2) IDPs are completed on a regular basis to facilitate career planning and development. With their DMD or CMD, mentees participate in team setup and take initiative and responsibility for identifying appropriate mentors. Mentees meet with potential mentors to determine compatibility, establish explicit goals including using their IDPs, and develop a mentoring meeting schedule. Each mentor team is expected to include an external mentor, outside of the mentee’s department with whom the mentee is less likely to have a potential financial or research conflict of interest (i.e., revenue, authorship, space) to provide career trajectory and/or work–life balance mentorship. To promote the bidirectional nature of mentoring and reward mentoring excellence, mentees supply evaluations that are included in their mentors’ advancement packet. Mentees are also advised on how to fill gaps in the mentoring team, such as how to identify an advocate or sponsor for their career development, usually a senior faculty or institutional leader [16,17].

## Initiative I: MA Workshops and Events

The opportunity to share best practices in mentoring with peers in the context of mentoring workshops was the earliest resource made available to our mentors. We employ a flexible approach to reach the large group of interested faculty mentors and mentees working in a busy academic–clinical organization by offering workshops during the work week at a variety of times (i.e., early morning, lunch time, and early evening) and the weekend. The curriculum consists of a core module of five workshops, based on the curriculum developed by Pfund et al. [7,10], modified by developing additional scenarios relevant to faculty from nonresearch tracks (e.g., clinical, teaching). Consistent with Pfund et al. [10] topics included: aligning expectations, including the use of IDPs; maintaining effective communication and delivering feedback to trainees; assessing your mentee’s understanding and knowledge; addressing diversity and inclusion; promoting professional development; and fostering independence. The number of workshop hours was reduced from the original eight to four with the topics compressed over the session time. Based on faculty feedback, another workshop was added to better prepare mentors to provide up to date advice on the intricacies of advancing through the University of California merit and promotion system. In addition, case studies are regularly updated to reflect new, timely topics in academic health.

We also developed a workshop for mentees on establishing successful mentorship [3]. Workshop topics include: identifying an appropriate mentor; initiating the mentoring process; avoiding conflict and establishing expectations; differences between mentoring and sponsorship as well as how to find a sponsor; and understanding the requirements for advancement in their track. Finally, we hold schoolwide speed mentoring events that bring senior mentors and junior faculty together to help identify new mentors.

### MA Workshop Participants and Measures

MA events have been attended by more than 500 SOH faculty, staff, and students. A total of 331 faculty have attended at least one of the 88 1-h MA workshops since 2011, representing approximately 46% of mid- and senior faculty. Table 1 shows the characteristics of MA participants. The average workshop attendee was 53 years of age with slightly more men (56%) participating. The race and ethnicity distribution of workshop participants has been representative of the general SOH faculty (Table 1).

**Table 1.** Characteristics of mentoring academy participants, 2014–2018 (n = 331)*

	*n*	Percent (%)
Gender (*n* = 330)		
Male	192	58
Female	138	42
Race (*n* = 285)		
White	218	76
Asian	37	12
Other race	15	5
Black or African American	11	5
Native American or Alaskan Native	4	2
Hispanic/Latino (*n* = 288)		
Yes	30	10
Degree (*n* = 300)		
MD	178	59
PhD	85	28
MD and PhD	22	7
Other	15	5
Faculty title (*n* = 238)		
Primarily research (tenured)		
Professor	51	21
Associate Professor	21	9
Primarily clinical (nontenured)		
Professor	43	18
Associate Professor	25	11
Both clinical and research (nontenured)		
Professor	34	14
Associate Professor	12	5
Research Professor (nonclinical)	13	5
Other including adjunct faculty (nontenured)	39	16

* Number in parenthesis is the number of participants for which data on the characteristic were available, either from human resources data or from the participant’s mentoring academy entry survey.

The majority of MA workshop participants (58%) completed online entry surveys prior to attending their first workshop that asked them to rate their current level of mentoring skills in several competency areas, based on surveys used in previous studies on mentoring [7,10]. Changes in perceived skill were assessed using the validated Mentoring Competency Assessment tool [18] with skills rated on a scale of 1 (not at all skilled) to 7 (extremely skilled). Questions were added using the same Likert format to the survey related to the training module we added focusing on understanding the promotion and merit procedure and ways for the mentor to assist the mentee in preparing for academic advancements. In addition, there was space on the survey for general comments about the workshop experience.

### MA Workshop Results

Table 2 shows the results of paired sample *t*-tests to evaluate perceived improvement in mentoring skills before and after participating in the workshops for prerating and postrating (*n* = 347 participants). Participants consistently rated their mentoring skills higher after each workshop despite the fact that they rated their preworkshop mentoring skills relatively high (i.e., preworkshop mean rating was greater than 4.0 on four of five topics covered). A composite score based on averaging presurvey (*M* = 4.47, SD = 1.11) and postsurvey (*M* = 5.44, SD = 0.97) ratings across the five workshop modules was also statistically significant (95% confidence interval = 0.81, 1.12; *p* < 0.0001). A majority of participants (92%) (328 of 355 responses) in the core workshops said they would recommend the MA workshops to their colleagues.

**Table 2.** Comparisons in perceived skill level before and after workshop participation

Workshop topic	Presurvey mean (SD)	Postsurvey mean (SD)	Mean change*	Sample size	95% confidence interval for mean difference
Aligning expectations and developing contracts	4.33 (0.95)	5.71 (0.80)	1.38	87	1.14, 1.63
Maintaining effective communication, delivering feedback, assessing understanding	4.86 (0.95)	5.57 (0.88)	0.71	96	0.48, 0.93
Addressing diversity and inclusion	4.51 (1.09)	5.18 (0.93)	0.67	57	0.42, 0.92
Promoting professional development and fostering independence	4.66 (1.04)	5.59 (0.88)	0.93	53	0.71, 1.15
Understanding faculty series, titles, and promotion portfolios at our institution	3.67 (1.22)	4.82 (1.19)	1.15	54	0.78, 1.52

* *p* < 0.0001 (1-tailed).

To facilitate an inductive content analysis, the authors created a database containing all postworkshop comments which were reviewed by each member of the group. The group then convened to develop a set of themes pertaining to workshop content, format, and overall satisfaction. Several themes emerged including that the participants found both the content and format of the MA workshops to be valuable and that they served to reinforce best practices for mentoring. Another common theme was the value of the workshops in delivering information on topics not covered elsewhere in the health system, such as how to mentor for diversity and inclusion (Supplementary Fig. 1 presents sample comments).

### MA Workshop Discussion

In this replication of implementing the curriculum from the Pfund et al. [7], we found similar results regarding self-ratings in improvement and likelihood in recommending the training in a group of faculty members who were more diverse in race, ethnicity, and a higher percentage of females than in the original report [7]. In keeping with our goal of aiming to include nonresearchers as well as researchers, we had a substantially higher rate of nonresearch physicians as participants than in Pfund et al. [7]. Our faculty rated themselves slightly less skilled before the training than participants in the original report [7], although the change in skill was rated as strong, and in some categories stronger, in our sample. Thus, the truncated format with fewer hours per topic in our delivery did not appear to weaken the effect of the training. This analysis does not include ratings by mentees on how well the mentor training may have affected their behavior toward their mentee; however, we expect that there would be a similar change in behavior given our findings closely mirror the positive change in self-ratings and the qualitative comments by participants in Pfund et al. [10] which was associated with changes in behavior.

## Initiative II: Career Development Planning Organized by the MA

Goal setting is recognized as a critical step in achieving one’s objectives in the work environment [19] and increasingly expected in the academic environment [9,20–22]*.* The use of IDPs as a formal tool to communicate career objectives between mentors and mentees is also increasing in academic settings [23], particularly with the National Institutes of Health 2014 [24] policy requiring IDPs for funded trainees. IDPs can serve several purposes [3] including: (1) an opportunity for self-reflection; (2) a “road map” for accomplishing goals; (3) identification of skill gaps, strengths, and weaknesses; (4) identification of barriers and resources; and (5) a formal process and document to create a shared understanding of goals between a mentee and mentor [9]. Research on the value of IDPs for postdoctoral trainees has shown that IDPs result in higher goal attainment [20]. However, literature on the use of IDPs for faculty success, particularly nonresearch faculty is lacking [22].

An initiative driven by the MA included the expectation for junior faculty (i.e., assistant professors, early associate professors) to annually complete their IDP with their mentors. Modest stipends are given to DMDs, when their department achieves a minimum of 60% of the faculty submitting their IDPs. DMDs with high rates of IDP submissions for their department (e.g., 85% or >) have received recognition at an annual dean’s award ceremony.

During this phase of the MA, templates for developing IDPs were made available to mentees, mentors, and DMDs. It was expected that the templates would be adapted to best meet the requirement of structured career planning between a mentor and mentee. The resulting IDPs typically included a section on goal setting, time distribution between roles (e.g., research, clinic, teaching, self-development, service), and fields for describing resources needed and challenges experienced. To better understand how IDPs were being used at our institution and to formulate some ways the MA could address common challenges, we examined the content of IDPs that were completed by health faculty from 25 departments and one research center during a 4-year period (2014–2018).

### IDP Review Methods

To facilitate an inductive content analysis, individually identifying information was removed by one author (REG) from paper copies of 222 IDPs completed by assistant professors (mentees) between 2014 and 2018. The number of IDPs represents approximately 65% of the 340 individuals with junior faculty (assistant or early associate rank) appointments during the time period. Ten IDPs were initially coded for mentions of supports and resources received. After review by the authorship group, all the IDP mentions were then entered into an Excel database. This served as the data source of the frequency of supports, resources, and barriers experienced and to summarize them into a set of salient themes. The primary fields in the final analytic database included the mentee’s self-reported: (a) career track and academic rank; (b) percent effort distribution (patient care, research, teaching, administration, self-development, or service); (c) constraints and resources to achieve their current activities; (d) barriers and/or resources to achieve new goals. We report here on the barriers and resources. The authors read through the full set of de-identified data and arrived at a set of summarized themes through group discussion. It should be noted that the analysis of IDPs was not intended to create a data set for research, but to serve as a potentially rich source of information to inform the development of resources to meet the needs of our junior faculty.

### IDP Review Findings

**Fig. 2.**
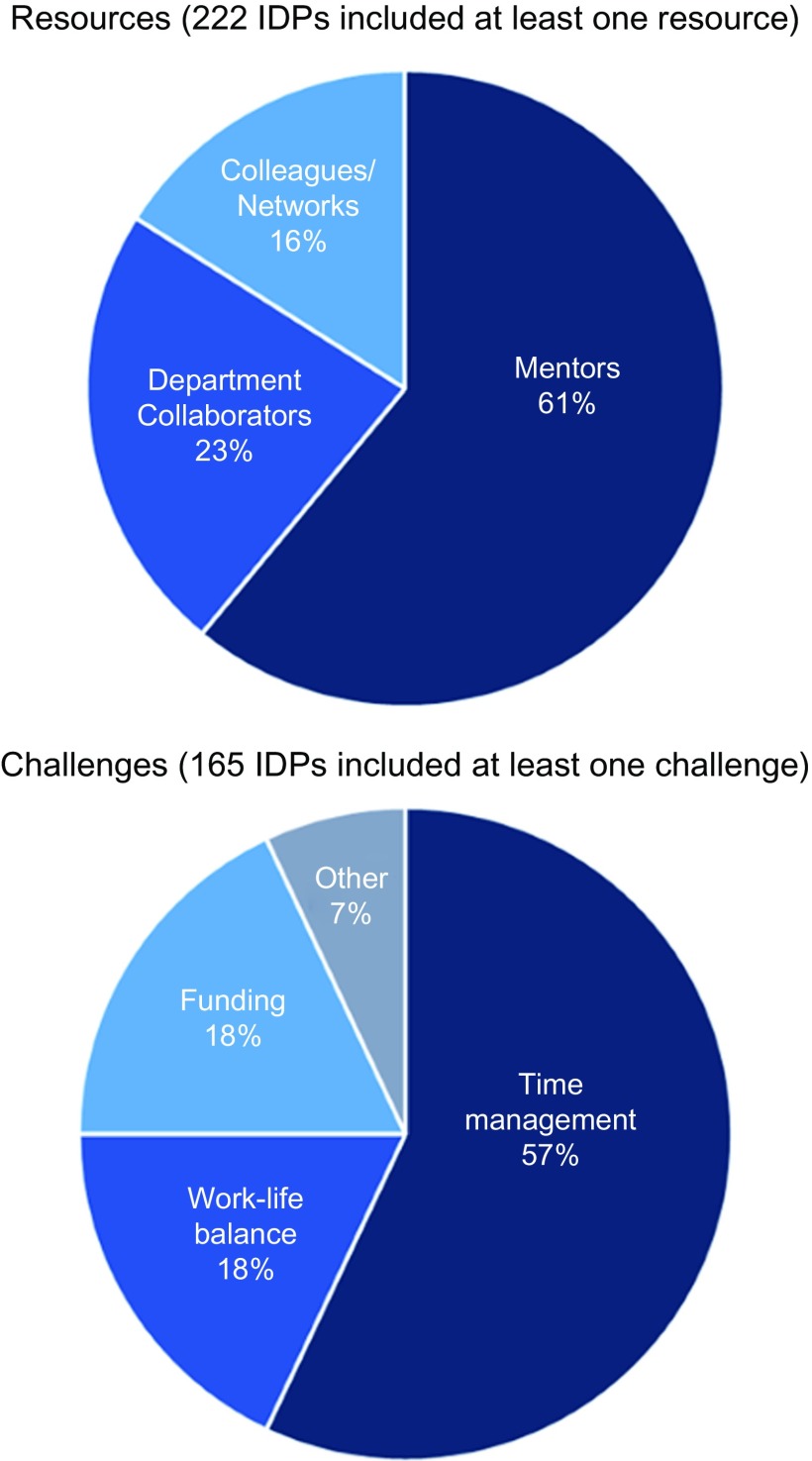
Resources and challenges for career goal achievement as reported in individual development plans (IDP) of 222 junior faculty, 2014–2018. IDP, individual development plans.

Junior faculty, regardless of employment track, most commonly noted their mentors and departmental collaborators as their primary resources for career development. Examples included help with time management and prioritizing workload to enable them to focus on their career and goals. Exposure to outside institutions and interdisciplinary teams was also frequently mentioned as a resource. Collaborators were noted for assisting in finding out about new funding opportunities to support research or career goal efforts. Finally, access to other clinicians, networking, and access to listservs emerged as themes related to career support (See Fig. 2.)

The majority of IDPs (74%) included reported challenges such as lack of time or difficulty with optimal time management. Specifically, junior faculty mentioned lacking protected time for research or other tasks and lacking the experience to select activities or tasks that would clearly benefit their career development and advance their goals. Achieving an optimal or healthy work–life balance was also frequently mentioned. Junior faculty noted in their IDPs that they had difficulties in achieving the self-care necessary to prevent burnout and having sufficient time and/or energy to dedicate to their family, especially if they had young children. Equally frequent were challenges related to limits in funding and lack of financial support, including insufficient funding to participate in career development activities and conferences. Other challenges cited were inadequate space, laboratory equipment, and experience/skill set (i.e., expertise, leadership skills, ability to delegate work) (See Fig. 2).

## General Discussion

Our experience suggests that a comprehensive mentoring program can be implemented to address the breadth of academic tracks present in traditional academic health centers. We found that many, but not all, of the concerns among mentors and mentees are common regardless of track. In order to resonate with faculty from the different tracks, we refined our curriculum and tools to include case studies reflecting potential challenges and characters across clinical, research, and teaching settings. We recommend that workshop content be updated on a regular basis to resonate with the changing demands of health system faculty. Findings from our analysis on our mentoring workshops suggest that while mentors’ self-ratings are fairly high on their preworkshop skills, intense, small group workshops can lead to perceived, significant improvement after workshop participation.

The findings from the IDP analysis reinforce the value that junior faculty place on robust mentoring and the need for a mentoring program to develop and strengthen mentoring skills. Furthermore, our junior faculty frequently noted the contribution of their peers as a mentoring resource. Thus, both junior faculty and their mentors should actively consider peer mentors as part of a mentoring team. There are many successful peer mentoring models [25,26], including the use of “mentoring circles,” a hybrid of peer-to-peer and mentoring networks that meshes senior mentors and peer mentors in a group setting [27].

The IDP analysis also suggests that mentors should be prepared to discuss and suggest resources and tips for helping junior faculty manage and prioritize their time. Work–life balance and self-care issues were another prevalent theme in the IDPs, not surprisingly, as these issues are already fairly well recognized as concerns in academic medicine [28–30]. Mentors who initiate discussions on these issues are likely to be appreciated by their junior faculty mentees.

Ultimately, many of the challenges raised by junior faculty may need to be addressed on a systems level by the institution. For example, leadership may not only ensure that the relevant workshops on time management are offered, but also provide release time for faculty members from their clinic or teaching responsibilities in order to attend the workshops. The need for on-site child care was one of the most common requests from our junior faculty, but can only be accomplished if leadership considers it a priority. Human resource administrators may also be able to assist with suggesting childcare resources that are shared during the faculty onboarding process and they could share those resources with mentors who are interested in mentoring faculty around work–life integration challenges. One example of low-cost, grass-roots resource is our recently initiated Facebook group for sharing information and reviews of childcare options, recreational activities for families, and baby-sitting co-ops.

The strength of this analysis is that it is one of the first to systematically review reports of resources and barriers in the context of goal attainment in junior faculty in an academic health setting. We recognize that our experience is representative of only one institution; however, our program is likely generalizable to other institutions with similar leadership support at comparably sized academic health institutions. We acknowledge the limitations in drawing inferences about how changes in perceived skills as rated by participants in the workshops might reflect changes in actual behavior; however, Pfund et al. [8,10] did detect improved mentoring behavior using the curriculum upon which we based our workshops. There are also limitations in our IDP analysis, including that that there may be bias in the analysis with those who completed the IDPs and answered questions about resources and barriers having stronger feelings about those issues than the average faculty member. Furthermore, because the IDPs were not completed anonymously, it is likely that some faculty members may not have been comfortable reporting the barriers they have encountered due to concern about how their supervisors may have reacted. The limits of our retrospective analysis should be addressed using a prospective analysis, incorporating a randomized control design to assess the effectiveness of a career planning tool on goal achievement, promotion, and retention. We are in the process of launching an electronic, web-based IDP system, with uniform fields that will provide an opportunity for prospective, finer-grained analyses. Ultimately, an assessment of many of the most critical outcome measures (e.g., retention and promotion) will require an extended period of time (years) to assess. Finally, we have yet to assess the effect of an “academy” versus a “mentoring program” on whether or not it has a more robust effect in changing the recognition and skill of mentoring at an institution. Future research between institutions on mentoring may be necessary to determine the value of an “academy” over a general mentoring program.

## Conclusion

Our MA can serve as a model for other academic health institutions. Such programs require strong, visible support from leadership to modify and sustain a mentoring culture [1] as well as input from junior faculty in order to maintain relevancy to current issues. Our program will hopefully serve as a model for other academic health systems to improve the quality of mentoring for faculty and trainees who across career faculty tracks in academic health systems.

## Supplementary material

For supplementary material accompanying this paper visit https://doi.org/10.1017/cts.2019.406. click here to view supplementary material
