# Supplementary figures and images for: Building a comprehensive mentoring academy for schools of health
Source: J Clin Transl Sci. 2019 Aug 28;3(5):211–7. doi: 10.1017/cts.2019.406 (PMC6813514; doi:10.1017/cts.2019.406)

**Supplemental Material**

**Figure 1. Sample comments from workshop participants.**

*
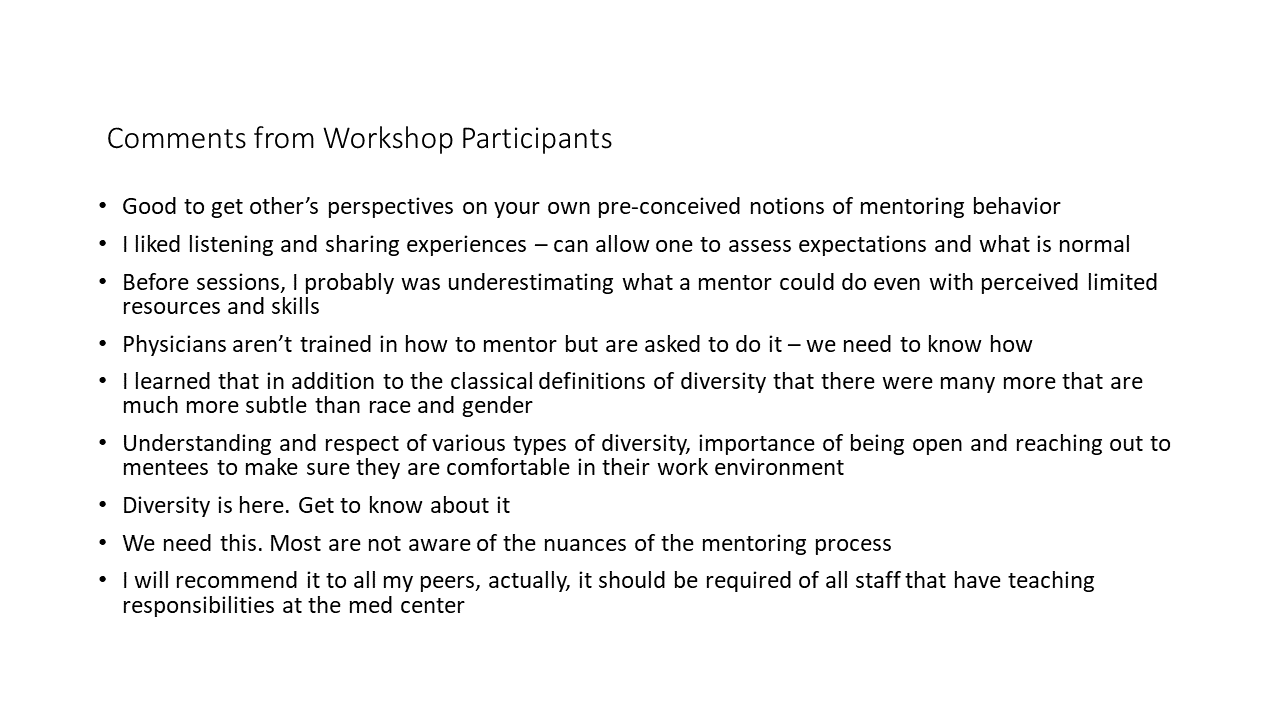
*

Supplement: Supplementary file 1 [file S2059866119004060sup001.docx]
